# *Angiostrongylus cantonensis* Infection of Central Nervous System, Guiana Shield

**DOI:** 10.3201/eid2406.180168

**Published:** 2018-06

**Authors:** Antoine L. Defo, Noémie Lachaume, Emma Cuadro-Alvarez, Chimène Maniassom, Elise Martin, Falucar Njuieyon, Fanny Henaff, Yajaira Mrsic, Annabelle Brunelin, Loic Epelboin, Denis Blanchet, Dorothée Harrois, Nicole Desbois-Nogard, Yvonne Qvarnstrom, Magalie Demar, Céline Dard, Narcisse Elenga

**Affiliations:** Andrée Rosemon Hospital, Cayenne, French Guiana (A.L. Defo, N. Lachaume, E. Cuadro-Alvarez, C. Maniassom, E. Martin, F. Njuieyon, F. Henaff, Y. Mrsic, A. Brunelin, L. Epelboin, D. Blanchet, M. Demar, N. Elenga);; Université de Guyane, Cayenne (L. Epelboin, D. Blanchet, M. Demar, N. Elenga);; Basse-Terre Hospital, Guadeloupe, French West Indies (D. Harrois);; University Hospital of Martinique, Fort-de-France, Martinique (N. Desbois-Nogard);; Centers for Disease Control and Prevention, Atlanta, Georgia, USA (Y. Qvarnstrom);; University Hospital of Grenoble-Alpes, Grenoble, France (C. Dard)

**Keywords:** Angiostrongylus cantonensis, nematodes, parasites, eosinophilic meningitis, transverse myelitis, Guiana Shield, meningitis/encephalitis

## Abstract

We report a case of eosinophilic meningitis complicated by transverse myelitis caused by *Angiostrongylus cantonensis* in a 10-year-old boy from Brazil who had traveled to Suriname. We confirmed diagnosis by serology and real-time PCR in the cerebrospinal fluid. The medical community should be aware of angiostrongyliasis in the Guiana Shield.

In September 2017, a previously healthy 10-year-old boy from Brazil came to the emergency department of Andrée Rosemon Hospital in Cayenne, French Guiana, a French territory that forms the Guiana Shield together with Guyana (formerly British Guiana), Suriname, and the Brazil state of Amapá. He related a 4-day history of helmet headache, repeated vomiting, and hyperthermia (38.5°C). The patient had lived in Saint-Laurent-du-Maroni, a city on the French Guiana border with Suriname, for 5 years and had recently returned from a 3-day trip in Suriname. He had no memory of ingesting slugs, snails, or uncooked vegetables, but he reported playing with snails during the rainy season (April–August). 

At admission to the pediatric department, he was afebrile with a good state of consciousness (Glasgow coma score 15). Our physical examination revealed a stiff neck, with positive Kernig and Brudzinski signs but no focal deficits. Hematology revealed a leukocyte count of 12.30 × 10^9^ cells/L (reference range 4–14.5 × 10^9^ cells/L) with 5.49 × 10^9^ eosinophils/L (reference range 0.05–0.85 × 10^9^ eosinophils/L). C-reactive protein was <3 mg/L; liver and renal function tests were normal. Computed tomography of the head showed unremarkable results. We performed a lumbar puncture; cerebrospinal fluid (CSF) analysis revealed 8.7 × 10^6^ leukocytes/L (30% neutrophils and 70% lymphocytes), protein 0.43 g/L, glucose 4.2 mmol/L, and lactates 2.2 mmol/L. Gram stain result was negative for bacteria. Results of India ink test and microscopic examination of CSF were negative for *Cryptococcus* spp. We saw no helminth larvae in the CSF. Serologic test results for *Treponema pallidum*, *Borrelia burgdorferi*, *Leptospira* spp., *Mycoplasma pneumoniae*, *Chlamydiophila pneumoniae*, *Brucella* spp., herpes simplex virus, and HIV were all negative. Microscopic examinations of 3 fecal specimens using the concentration method and Baermann technique showed negative results. We began empiric treatment with intravenous cefotaxime (300 mg/kg/d).

On day 6 of hospitalization, paraparesis of the lower limbs (more marked on the left) and dysuria appeared; meningeal syndrome persisted. A cerebromedullary magnetic resonance imaging (MRI) scan revealed myelitis lesions through a marrow signal abnormality ranging from T2 to T10 and a discrete signal enhancement after gadolinium injection (Figure, panel A). Electroencephalography results were unremarkable. We performed a second lumbar puncture on day 7; CSF showed 5.5 × 10^6^ leukocytes/L with 92% eosinophils, protein 0.42 g/L, glucose 2.80 g/L, and lactates 2.7 mmol/L. Results of CSF bacterial cultures and PCRs for herpes simplex virus and enterovirus were negative. Serologic testing by Western blot was negative for *Gnathostoma* spp. nematodes but positive for *Angiostrongylus* spp. roundworms by detection of the specific 31-kDa antigenic band (*1*). Diagnosis of angiostrongyliasis was confirmed by *A. cantonensis* DNA detection in the CSF by real-time PCR performed by the US Centers for Disease Control and Prevention (Atlanta, GA, USA) (*2*). 

**Figure:**
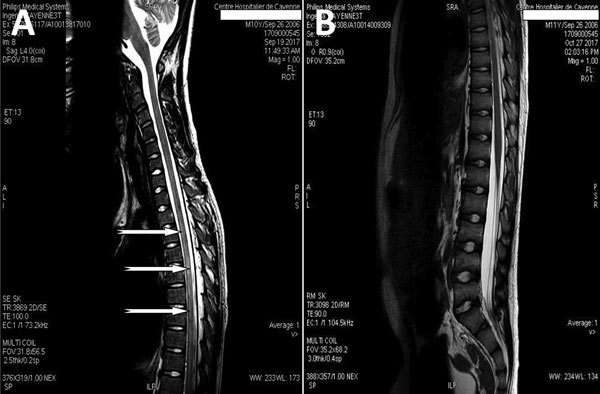
Magnetic resonance imaging (MRI) of the spine in a 10-year-old boy from Brazil with *Angiostrongylus cantonensis* infection. A) MRI before treatment showing myelitis; sagittal T1 postcontrast sequences show intramedullary enhancement in the thoracic spinal cord T2–T10 with diffuse leptomeningeal enhancement (arrows). B) Normal MRI 1 month after treatment.

We treated the patient with oral ivermectin (200 μg/kg/d for 10 days) in combination with intravenous methylprednisolone (30 mg/kg/d for 5 days), followed by oral prednisolone (2 mg/kg/d), which was gradually discontinued over 1 month. The patient’s condition improved noticeably, with progressive disappearance of headaches, dysuria, and paraparesis in the following weeks. A cerebromedullary MRI performed on day 38 after admission showed almost complete recovery from the anomalies detected previously and did not report new anomalies (Figure, panel B). Three months after the onset of the disease, the patient had recovered completely without any sequelae.

Our findings demonstrate the presence of *A. cantonensis* roundworms in the Guiana Shield, in the context of a recent emergence of angiostrongyliasis in Brazil (*3*), the Caribbean region (including other French territories of the Americas) (*4*,*5*), and the southern United States (*6*,*7*). The frequency of *A. cantonensis* infections in humans in the Guiana Shield is probably underestimated as a result of the spontaneous course of recovery for most cases (*8*), lack of knowledge of the parasite by health professionals, limited availability of laboratory diagnostic tools, and the absence of national surveillance. Although the disease usually resolves spontaneously, case-fatality rates can reach 5% (*9*). The lack of clinical suspicion for angiostrongyliasis on the basis of signs and symptoms and delay in initiation of treatment may lead to adverse neurologic outcomes, especially in young children (*10*). Because the patient in this study had traveled to Suriname shortly before symptom onset, the country of origin of the infection could not be determined. The likely route of transmission was contact with a contaminated mollusk, such as the giant African snail *Achatinafulica fulica*, which is a new and invasive species in Latin America and a known vector for *A. cantonensis* roundworms*.* Our case illustrates the necessity for healthcare providers to consider angiostrongyliasis in cases of eosinophilic meningitis in the Guiana Shield, especially in young children.
